# Retinal Hemorrhages and Long-Term Ocular Outcomes in Neonatal Hypoxic-Ischemic Encephalopathy

**DOI:** 10.3390/medicina61050906

**Published:** 2025-05-16

**Authors:** Emrah Utku Kabataş, Seda Aydoğan, Ahmet Alp Bilgiç, Nurdan Dinlen Fettah, Naciye Kabataş, Dilek Dilli, Ayşegül Zenciroğlu

**Affiliations:** 1 Department of Ophthalmology, University of Health Sciences, İzmir City Hospital, 35170 İzmir, Turkey; 2 Department of Neonatology, University of Health Sciences, Etlik City Hospital, 06170 Ankara, Turkey; drsedakunt@gmail.com (S.A.); nrdinlen@gmail.com (N.D.F.); dilekdilli2@yahoo.com (D.D.); azenciroglu@hotmail.com (A.Z.); 3 Department of Ophthalmology, University of Health Sciences, Ulucanlar Eye Hospital, 06250 Ankara, Turkey; ahmetalp55@gmail.com; 4 Department of Ophthalmology, Ekol Hospital, 35630 İzmir, Turkey; aktasnaciye@yahoo.com

**Keywords:** hypoxic ischemic encephalopathy, newborn, retinal hemorrhage, refractive errors, Roth spots, strabismus

## Abstract

*Background and Objective:* This study aims to investigate the clinical significance and risk factors of retinal hemorrhages (RH) and white-centered retinal hemorrhages (Roth spots, RS) in neonates with hypoxic-ischemic encephalopathy (HIE), as well as their long-term ophthalmologic outcomes. *Materials and Methods:* Neonates diagnosed with HIE were classified into three stages according to the Sarnat classification. A comprehensive ophthalmologic assessment was performed within the first three days of life and at two years of age. Retinal hemorrhages were staged based on the Egge classification, and the presence of RS was also documented. The clinical characteristics and risk factors associated with RH and RS were systematically recorded. *Results:* Retinal hemorrhages were identified in 178 eyes (42.3%), and RS were observed in 180 eyes (42.8%). The prevalence of both RH and RS was significantly higher in neonates with Stage 2 and Stage 3 HIE (*p* < 0.001). The resolution time for both RH and RS was significantly prolonged in neonates with Stage 3 HIE compared to those with lower grades (*p* < 0.001). Furthermore, the frequency of grade 3 RH increased with advancing HIE stages (*p* < 0.001). Logistic regression analysis revealed that Stage 2 HIE (OR: 5.41, 95% CI: 1.19–24.54, *p* = 0.03) and Stage 3 HIE (OR: 27.17, 95% CI: 5.38–137.25, *p* < 0.001) were significantly associated with RS. Similarly, Stage 2 HIE (OR: 4.54, 95% CI: 1.00–20.68, *p* = 0.05) and Stage 3 HIE (OR: 40.88, 95% CI: 7.75–215.68, *p* < 0.001) were significantly associated with RH. At the age of two, strabismus was identified in 34 (18.4%) patients, while refractive errors were detected in 68 (37.4%) patients. *Conclusions:* The prevalence of RH and RS increases in correlation with the severity of HIE. While these hemorrhages generally resolve spontaneously, the risk of refractive errors and strabismus remains elevated.

## 1. Introduction

Retinal hemorrhages (RH) require long-term follow-up due to their potential impact on vision. The etiology of these hemorrhages varies across different age groups [1]. In older adults, the most common causes include diabetes mellitus, hypertension, and retinal vein occlusion [1]. However, data regarding the etiology of RH in the neonatal period remain limited in the literature. Traumatic delivery, the use of forceps or vacuum extraction, and perinatal asphyxia have been identified as potential risk factors [2,3,4,5,6,7].

Roth spots (RS) are white-centered superficial RH that were initially considered pathognomonic for infective endocarditis [8]. However, subsequent studies have demonstrated their presence in a variety of other pathological conditions, including leukemia and shaken baby syndrome [8,9,10,11]. The literature on RS in the neonatal period remains limited, with existing reports primarily describing their occurrence in otherwise healthy neonates following complicated deliveries [2,5].

Neonatal hypoxic-ischemic encephalopathy (HIE) is a severe neurological disorder that primarily manifests at birth or shortly thereafter, particularly in late preterm and term neonates [12]. This hypoxic-ischemic insult can compromise the retinal vasculature, potentially resulting in RH [13]. Persistent RH may lead to irreversible visual impairment in affected infants.

Despite the potential impact on the visual field, RH and RS in neonates with HIE have not been extensively investigated. Accordingly, this study aims to evaluate the clinical significance and potential risk factors of these hemorrhages, while also investigating the ophthalmologic outcomes of the affected patients at the age of two.

## 2. Materials and Methods

A retrospective review was conducted of the medical records of neonates diagnosed with HIE who were admitted to the neonatal intensive care unit of our hospital between January 2012 and December 2018 and subsequently referred to our clinic for ophthalmic evaluation by a neonatologist. The study protocol was approved by the local ethics committee (approval number: 2012-KAEK-15/1739) and adhered to the principles outlined in the Declaration of Helsinki. Written informed consent was obtained from the parents of all participants prior to the ophthalmological examination.

Patients were stratified into three groups based on Sarnat staging: Stage 1 HIE, Stage 2 HIE, and Stage 3 HIE [14,15]. Stage 1 was characterized by mild encephalopathy, including hypervigilance, sympathetic overactivity, and a normal electroencephalogram (EEG). Stage 2 encompassed moderate encephalopathy, marked by hypotonia, multifocal seizures, and an EEG demonstrating periodic or sustained delta activity. Stage 3 represented severe encephalopathy, in which affected neonates exhibited stupor and hypotonia, with EEG findings showing isoelectric or periodic activity [14,15]. Therapeutic hypothermia remains the sole established treatment for HIE and is administered to infants classified as Stage 2 HIE or higher [15].

Exclusion criteria included the presence of one or more of the following conditions: retinopathy of prematurity (ROP), congenital anomalies involving one or more organ systems, any intrauterine infections, and cases with incomplete or insufficient medical records.

All neonates diagnosed with hypoxic-ischemic encephalopathy (HIE) underwent a comprehensive ophthalmologic assessment within the first three days of life and at the age of two. In newborns, pupillary dilation was achieved using 0.5% tropicamide and 2.5% phenylephrine eye drops, administered three times at 10 min intervals. Following adequate dilation, 0.5% proparacaine HCl eye drops were applied for topical anesthesia, and a pediatric speculum was used to maintain eyelid opening. Retinal imaging was conducted using an indirect ophthalmoscope (Heine, Germany) with a 28-diopter lens or RetCam III system. All fundus examinations were conducted by the same ophthalmologist using a scleral depressor.

Retinal hemorrhages were classified into three grades according to the classification proposed by Egge et al. [16]. Grade 1 RH were defined as small hemorrhages within a limited area, characterized by fine linear bleeding confined to the peripapillary region. Grade 2 RH included a slightly larger extent of hemorrhage, with patchy, flame-shaped bleeding extending over an area not exceeding the diameter of the optic disc. Grade 3 RH were characterized by hemorrhages exceeding the optic disc diameter, accompanied by flame-shaped bleeding along the retinal vasculature and macular involvement. As Roth spots are not incorporated into this classification system, they were documented separately when observed.

Follow-up examinations were conducted on a weekly basis until complete resolution of RH was achieved. Additionally, a comprehensive ophthalmological examination was performed on all children at the age of two years. To assess refractive errors at two years of age, measurements were conducted 50 min after the administration of two drops of cyclopentolate (1%) at 5-min intervals using a handheld autorefractometer (Welch Allyn; Sure Sight Autorefractor, Skaneateles, NY, USA). The spherical equivalent (SE) was calculated for all eyes using the following formula: SE = sphere + 1/2 cylinder. All astigmatism values were recorded in plus cylinder notation. Ametropia was defined as SE refraction of ≥+2.5 diopters (D) or ≤−0.5 D in either eye, or astigmatism of ≥1.5 D.

The study evaluated patient demographics, including gestational age, birth weight, gender, and mode of delivery, along with the frequency and severity of RH, the prevalence of RS, the time to resolution of RH and RS, and the correlation between these parameters and the stage of HIE. Additionally, at the age of two years, the presence of refractive errors and strabismus was documented according to the severity of hypoxic-ischemic encephalopathy.

Statistical analyses were conducted using SPSS 20.0 (SPSS, Chicago, IL, USA). The Kolmogorov–Smirnov test was used to assess the normality of data distribution. Continuous variables were expressed as mean (standard deviation, SD). Comparisons between two groups were performed using Student’s *t*-test or the Mann–Whitney U test, while categorical variables were analyzed using chi-square tests. For comparisons involving three groups, a one-way analysis of variance (ANOVA) was conducted. Multivariate analysis was performed by incorporating potential confounding variables to identify risk factors associated with RH and RS. Odds ratios (ORs) with corresponding 95% confidence intervals (CIs) were reported. A two-tailed *p*-value of <0.05 was considered statistically significant.

## 3. Results

A total of 420 eyes from 210 neonates were included in the study. Group 1 consisted of 32 patients with Stage 1 HIE, Group 2 comprised 129 patients with Stage 2 HIE, and Group 3 included 49 patients with Stage 3 HIE. There were no statistically significant differences in gestational age, gender, or birth weight among the groups (*p* = 0.354, *p* = 0.144, and *p* = 0.340, respectively). The demographic characteristics of the study population are summarized in Table 1.

**Figure 1:**
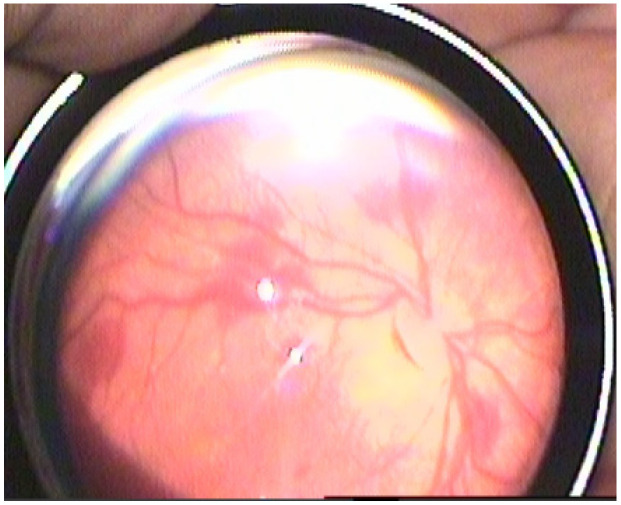
Grade 2 retinal hemorrhage.

**Figure 2:**
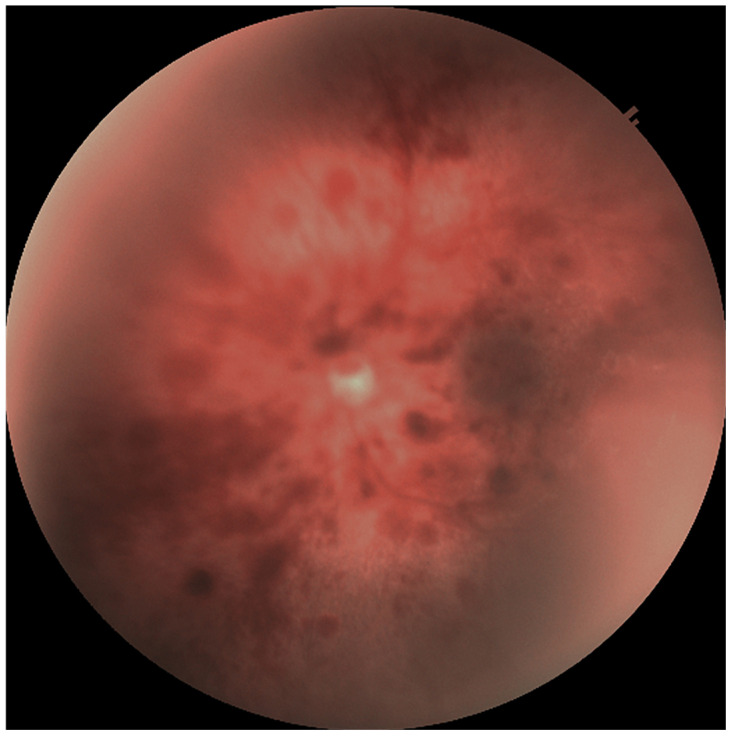
Grade 3 retinal hemorrhage.

**Figure 3:**
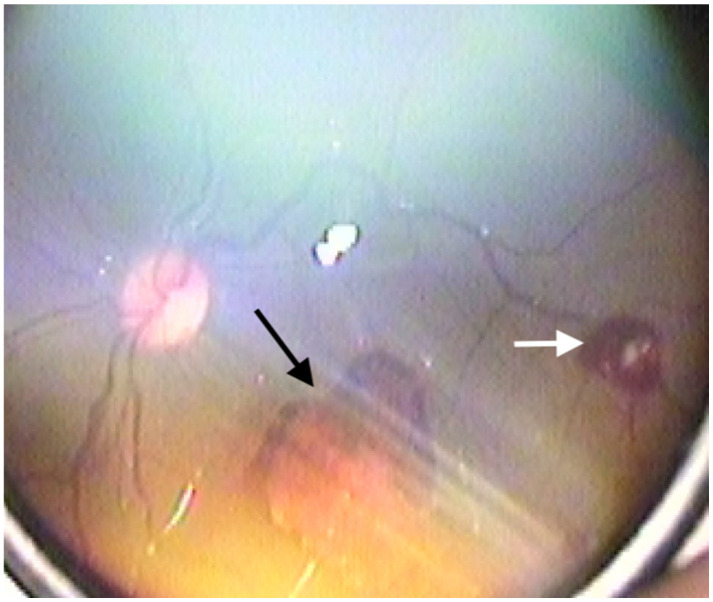
Grade 3 retinal hemorrhage (black arrow) and Roth spot (white arrow).

Retinal hemorrhages were identified in 178 eyes (42.3%), while RS were observed in 180 eyes (42.8%). The prevalence of both RH and RS was significantly higher in neonates with Stage 2 and Stage 3 HIE (*p* < 0.001). Examples of retinal hemorrhages and Roth spots are presented in Figure 1, Figure 2 and Figure 3. The mean resolution time for RH and RS was 20.21 ± 6.19 days and 13.3 ± 2.2 days, respectively. Notably, the resolution time for RH and RS was significantly prolonged in the Stage 3 HIE group compared to the other groups (*p* < 0.001). Moreover, an increasing HIE stage was associated with more severe RH, as classified by the Egge classification system. Detailed data regarding RH are presented in Table 2. Importantly, all instances of RH and RS were bilateral.

In the logistic regression analysis, the presence of RH and RS was considered the dependent variable, while gestational age, birth weight, mode of delivery, and HIE stage were included as independent variables. The analysis revealed that only Stage 2 HIE (OR: 5.41, 95% CI: 1.19–24.54, *p* = 0.03) and Stage 3 HIE (OR: 27.17, 95% CI: 5.38–137.25, *p* < 0.001) were significantly associated with RS. Similarly, only Stage 2 HIE (OR: 4.54, 95% CI: 1.00–20.68, *p* = 0.05) and Stage 3 HIE (OR: 40.88, 95% CI: 7.75–215.68, *p* < 0.001) were significantly associated with RH.

A total of 182 patients underwent a follow-up examination at the age of two years. The distribution of refractive errors and strabismus status among these patients is presented in Table 3. Of these patients, 114 (62.6%) were emmetropic, 30 (16.5%) had hyperopia, 10 (5.5%) had myopia, and 28 (15.4%) had astigmatism. The spherical equivalent (SE) values ranged from −3.00 D to +5.00 D, while cylindrical values varied between +0.50 D and +3.00 D.

Strabismus was identified in 34 patients (18.7%), with 31 (17%) presenting with exotropia and 3 (1.6%) with esotropia. Four patients in the Stage 3 HIE group were diagnosed with cortical blindness. Patients with cortical blindness exhibited total optic atrophy and searching nystagmus. No posterior segment pathology was detected in the other patients.

## 4. Discussion

Retinal hemorrhages are a common ophthalmic finding in healthy neonates and may serve as crucial diagnostic and prognostic indicators of systemic hypoxia-related conditions [6,12,13,14]. Retinal hemorrhages have the potential to cause vision loss [1]; therefore, their timely diagnosis, follow-up, and management are of paramount importance, particularly given the challenges of assessing visual impairment in the neonatal period.

Previous studies have reported varying prevalence rates of RH in healthy neonates. Emerson et al. [2] identified a prevalence of 34%, Hughes et al. [3] reported 34%, Zhao Qi et al. [4] found 24.5%, Callaway et al. [5] reported 20.3%, and Yanli Z et al. [7] observed a rate of 39.36%. These studies have generally linked RH to vaginal delivery and instrumental-assisted births, such as forceps delivery. However, in contrast to these findings, our study revealed that RH was more frequently observed in neonates with higher stages of hypoxic-ischemic encephalopathy (HIE), and cesarean section (C/S) was the more common mode of delivery in this group. This finding suggests that the neonates with HIE in our cohort may have undergone a prolonged hypoxic and challenging antenatal course, necessitating delivery via C/S.

Existing literature indicates that hypoxic-ischemic events occur antenatally in approximately 20% of neonates, intrapartum in 30%, both before and during birth in 35%, and postnatally in only 10% of cases [17].

Another significant cause of RH is hypoxia-inducing conditions such as hypoxic-ischemic encephalopathy (HIE). Hypoxia, particularly when it leads to intracranial involvement, may contribute to RH development [18]. These hemorrhages are likely attributable to increased intracranial pressure resulting from impaired autoregulation due to hypoxic cerebral vasodilation, which subsequently elevates retinal venous pressure [18]. Moreover, hypoxia and asphyxia have been associated with early-onset neonatal thrombocytopenia, predisposing neonates to hemorrhagic complications [6].

Eris et al. reported a high incidence of RH (76.9%) in infants with HIE who underwent whole-body cooling [13]. Their study also found RH in 20.6% of neonates with Stage 1 HIE and in 76.9% of those with Stage 2–3 HIE [13]. Similarly, another study identified RH in 29.3% of neonates with HIE, although no significant correlation was observed between RH frequency and HIE severity [19]. Additionally, Quinglan Pu et al. demonstrated that asphyxia increased the likelihood of RH by 2.49-fold [20]. In our study, the overall prevalence of RH in neonates with HIE was 42.3%. We also observed that as the HIE stage increased, the severity of RH, according to the Egge classification, also increased. Furthermore, logistic regression analysis identified higher HIE stages as independent risk factors for retinal hemorrhage development.

Roth spots are superficial retinal hemorrhages located in the nerve fiber layer, characterized by a central white dot. They result from capillary rupture, leading to hemorrhage with a central fibrin-platelet cluster [8]. The underlying mechanisms of capillary damage include hypoxia, trauma, and inflammation. While RS have historically been associated with infective endocarditis [9], they have also been identified in various other systemic and ophthalmological conditions, including leukemia and shaken baby syndrome [10,11]. In our study, RS were observed in 42.8% of neonates with HIE. Moreover, logistic regression analysis identified higher HIE stages as independent risk factors for RS development. The increasing prevalence of RS with worsening ischemia underscores the impact of ischemia and hypoxia on the retinal vascular structure in HIE. The presence of RS in neonates with HIE highlights the importance of comprehensive retinal examinations in neonates with systemic ischemic conditions, as these findings may provide valuable prognostic information.

Callaway et al. reported that RS occurred in 30.2% of healthy neonates [5]. Additionally, Emerson et al. identified RS in healthy newborns, though their study did not specify prevalence rates [2].

There are limited data in the literature regarding the resolution time of RS and RH. Eris et al. reported that retinal hemorrhages involving the macula resolved within 38.57 ± 6.29 days, whereas those not involving the macula resolved within 24.27 ± 7.78 days [13]. Another study found that 86% of retinal hemorrhages resolved within two weeks, and no retinal hemorrhages were detected at four weeks [2]. In our study, the mean resolution time of RS was 13.3 ± 2.2 days, while the mean resolution time of RH was 20.21 ± 6.19 days. Notably, the hemorrhages with the longest resolution times were grade 3 hemorrhages. When comparing HIE stages, a significant difference was observed in the resolution time of both RS and RH (*p* = 0.004, *p* < 0.001, respectively).

Previous studies have suggested that lower gestational age may be a predictor of RH [5]. However, our study did not find a significant relationship between gestational age and RH.

In our study, refractive errors were identified in 37.4% of the participants, with 30 (16.5%) children diagnosed with hyperopia, 10 (5.5%) with myopia, and 28 (15.4%) with astigmatism. However, previous studies have reported similar [21], lower [22], or higher [23] prevalence rates of refractive errors. These discrepancies may be due to differences in ethnic backgrounds, the age at which refractive assessment was conducted, and the threshold values used to define refractive errors.

In this study, the prevalence of strabismus was found to be 18.7%, which is higher than the reported prevalence in healthy children (1.5–2.8%) [24,25]. However, previous studies have reported similar [21], lower [22], or higher [23] prevalence rates of strabismus. Additionally, we observed a significant increase in the prevalence of strabismus with advancing HIE stages (*p* = 0.005). One possible explanatory hypothesis suggests that adverse intrauterine and perinatal events occurring during a critical period of brain development may disrupt the prenatal programming of eye movement coordination and control [26].

An important aspect of our study is the identification of RS and RH in neonates with HIE. These findings suggest that the pathophysiology of RS may differ between neonates and older children, with hypoxia playing a more prominent role in neonates. This highlights the clinical significance of retinal examinations in neonates with systemic conditions such as HIE, as RS may serve as an indicator of both retinal and systemic ischemia. In neonates with RH, short-term visual symptoms—such as blurred vision, visual field defects, or distortions—cannot be assessed, underscoring the necessity of early screening during the neonatal period for timely detection. Moreover, in advanced-stage HIE, retinal hemorrhages may persist for an extended duration and should therefore be considered in the differential diagnosis of growing term infants.

### Limitations

Our study has several limitations that must be acknowledged. The retrospective design inherently restricts the generalizability of our findings, necessitating prospective studies to validate these results. Furthermore, future research should integrate advanced imaging modalities, such as optical coherence tomography (OCT), to achieve a more comprehensive characterization of RS and other retinal abnormalities in neonates. Another limitation is the absence of maternal factors assessment, such as maternal age and systemic conditions, which may have influenced the observed outcomes. Additionally, the lack of visual acuity and visual field assessments at two years of age represents a further limitation, as cooperation in this age group is generally suboptimal.

## 5. Conclusions

The prevalence of RH and RS increases with the severity of HIE. While these hemorrhages generally resolve without long-term sequelae, their resolution time tends to be prolonged as HIE severity increases. Therefore, we recommend that ophthalmologic examinations in these patients be conducted with particular attention to the potential presence of refractive errors and strabismus, even after the resolution of retinal hemorrhages. Further research is necessary to validate our findings.

## Figures and Tables

**Table 1:** Demographic data of the patients.

	Stage I HIE	Stage II HIE	Stage III HIE	*p* Value
**Gender n (%)**				0.354
**Female**	16 (50%)	54 (42%)	26 (53%)	
**Male**	16 (50%)	75 (58%)	23 (47%)	
**Gestational age (mean ± SD) (week)**	39.25 ± 0.84	38.89 ± 1.66	38.53 ± 1.91	0.144
**Birth weight (mean ± SD) (gram)**	3200 ± 148	3181 ± 663	3039 ± 626	0.340
**Delivery Type n (%)**				<0.001
**Vaginal delivery**	24 (75%)	76 (59%)	16 (33%)	
**Caesarean section**	8 (25%)	53 (41%)	33 (67%)	

**Table 2:** Details of the ophthalmologic examination of neonates with retinal hemorrhage and Roth spots.

	Grade I HIE	Grade II HIE	Grade III HIE	*p* Value
**Retinal haemorrhage (n = eye) n (%)**				<0.001
**+**	4 (6.2%)	92 (35.7%)	82 (83.7%)	
**−**	60 (93.8%)	166 (64.3%)	16 (16.3%)	
**Roth spots (n = eye) n (%)**				<0.001
**+**	4 (6.2%)	100 (38.8%)	76 (77.6%)	
**−**	60 (93.8%)	158 (61.2%)	22 (22.4%)	
**Resolution time of RH (day) (mean ± SD)**	10.50 ± 4.94	16.43 ± 3.67	24.92 ± 4.96	<0.001
**Resolution time of RS (day) (mean ± SD)**	10.50 ± 4.94	12.88 ± 2.59	14.18 ± 1.13	0.004
**Egge Classification (n = eye) n (%)**				<0.001
**Grade 1**	4 (100%)	14 (15.2)	-	
**Grade 2**	-	56 (60.9)	32 (39%)	
**Grade 3**	-	22 (23.9)	50 (61%)	

**Table 3:** Ophthalmic findings of the patients at two years of age.

	Grade I HIE (n = 22)	Grade II HIE (n = 117)	Grade III HIE (n = 43)	*p* Value
**Spherical equivalent, Diopters, (mean ± SD)**	1.6 ± 1.8	1.8 ± 1.3	1.8 ± 1.3	0.798
**Astigmatism, Diopters, (mean ± SD)**	1.6 ± 0.9	1.3 ± 0.8	1.4 ± 0.9	0.384
**Emmetropia n (%)**	10 (45.5%)	76 (65%)	28 (65.1%)	0.424
**Hyperopia n (%)**	5 (22.7%)	20 (17.1%)	5 (11.6%)	
**Myopia n (%)**	3 (13.6%)	5 (4.3%)	2 (4.7%)	
**Astigmatism n (%)**	4 (18.2%)	16 (13.7%)	8 (18.6%)	
**Strabismus n (%)**	1 (4.5%)	18 (15.4%)	15 (34.9%)	0.005
**Esotropia**	-	3 (2.6%)	-	
**Exotropia**	1(4.5%)	15 (12.8%)	15 (34.9%)	

## Data Availability

The original contributions presented in this study are included in the article. Further inquiries can be directed to the corresponding author.
